# Motor Nerve Conduction Block Predicting Outcome of Guillain-Barre Syndrome

**DOI:** 10.3389/fneur.2018.00399

**Published:** 2018-06-01

**Authors:** Jingwen Niu, Mingsheng Liu, Qing Sun, Yi Li, Shuang Wu, Qingyun Ding, Yuzhou Guan, Liying Cui

**Affiliations:** Department of Neurology, Peking Union Medical College Hospital, Chinese Academy of Medical Sciences, Beijing, China

**Keywords:** Guillain-Barre syndrome, acute inflammatory demyelinating polyradiculoneuropathy, acute motor axonal neuropathy, conduction block, functional outcome, Hughes functional grading scale

## Abstract

**Introduction:** Motor nerve conduction blocks (CBs) could be detected in both acute inflammatory demyelinating polyradiculoneuropathy (AIDP) and acute motor axonal neuropathy (AMAN). We aimed to identify the correlation between CBs and functional outcome in the two subtypes of GBS.

**Methods:** Motor nerve conduction studies were performed in 17 patients with AIDP and 23 with AMAN. All patients were treated with intravenous immunoglobulin, and their disabilities were evaluated with Hughes functional grading scale before treatment, 1 month and 6 months after onset.

**Results:** AMAN with CBs had higher reduction of Hughes grade (indicating more improved outcomes) at 1 month (1.71 ± 0.83 vs. 1 ± 0.67, *p* = 0.034) than AIDP with CBs. AMAN with CBs had higher reduction of Hughes grade at 1 month (1.71 ± 0.83 vs. 0.56 ± 0.73, *p* = 0.002) than AMAN without CBs. The reduction of Hughes grade at 1 month showed no significant difference between AIDP with and without CBs.

**Discussion:** Motor nerve CBs in AMAN indicated better prognosis than in AIDP.

## Introduction

Guillain-Barre syndrome (GBS) is currently the most frequent cause of acute flaccid paralysis worldwide. Although it generally follows a monophase course, up to 20% of patients remain severely disabled and approximately 5% die, despite immunotherapy (1). GBS consists of two major subtypes, acute inflammatory demyelinating polyradiculoneuropathy (AIDP) and acute motor axonal neuropathy (AMAN) (2). Conduction block (CB) is one of the most common electrophysiological features in both subtypes (3), with different pathophysiological mechanisms. In AMAN, CB could be reversible or followed by axonal degeneration (4). Some previous works have looked at the relationship between CB and poor prognosis in all types of GBS, and found no relationship (5, 6), while other works found that CB of the common peroneal nerve was a good dichotomizing parameter to identify a group of GBS patients at very low risk for developing respiratory failure (7). In this study, we aimed to identify the correlation between CB and the functional outcome in patients with AMAN and AIDP.

## Materials and methods

### Subjects

We prospectively collected GBS patients admitted to our hospital between 2011 and 2017. Patients conformed with the criteria of GBS defined by Asbury and Cornblath et al. (8), and with a duration <20 days were included. All patients were treated with intravenous immunoglobulin (IVIG) 0.4 g/kg/day for 5 days. Four patients were given a second round of IVIG treatment within 1 month, due to severe disability and little improvement after the first round. All patients received rehabilitation therapy. Hughes functional grading scale was evaluated before treatment, 1 month and 6 months after onset. Since no patient aggravated after treatment, clinical nadir was defined as the time at admission. Rapid recovery was defined as an improvement by two or more Hughes grades within 1month after onset, and slow recovery as the inability to walk independently (grade 3 or more) 1 months after neurological onset. All patients gave written informed consent according to the study protocol, which was approved by the Peking Union Medical College hospital institutional review board, in accordance with the Declaration of Helsinki.

### Electrophysiology

Electromyography (EMG) and Nerve conduction studies (NCSs) were performed with a CareFusion Nicolet EMG machine. The mean duration from onset to EMG and NCS was 13.2 days. Motor NCSs were performed in all subjects on the median, ulnar, fibular, and tibial nerves with percutaneous supramaximal nerve stimulation while recording the CMAPs with 10-mm disk electrodes. Detailed methods were described in previous studies (9). Bilateral nerves were studied in most cases. Orthodromic sensory NCSs were performed on median, ulnar, tibial, and fibular nerves. Conventional needle EMG was performed in abductor pollicis brevis, abductor digiti minimi, and tibialis anterior. The room temperature was maintained to ensure that the patients' skin temperature remained above 31°C. The diagnoses of CB and probable CB were made according to the criteria proposed by American Association of Neuromuscular and Electrodiagnostic Medicine (AANEM). Definite partial CB was defined as an amplitude decrement of more than 50% (60% for tibial nerve) with <30% temporal dispersion. Probable partial CB was defined as an amplitude decrement of 40–49% (50–59% for tibial nerve) with <30% temporal dispersion (10). In order to include only true CB, distal (compound motor action potential) CMAP had to be ≥1 mV. We analyzed serial NCSs of all patients and classified them into AIDP, AMAN, Equivocal, or normal according to electrodiagnostic criteria described by Rajabally et al. (2). Furthermore, based on changes in serial recordings, CBs in AMAN were classified into two groups; reversible CB and length-dependent CB. Reversible conduction failure was defined as CB being resolved quickly (2–4 weeks) with no development of excessive temporal dispersion or other demyelination features. The definition was in consistence with the criteria of early reversible conduction failure raised by Chan et al. (11). Length-dependent CB was defined as the disappearance of CB due to progressive reduction of distal CMAP amplitude. AMAN without CB was defined as typical AMAN (4).

### Statistical analyses

Descriptive statistics were presented as the mean ± standard deviation for continuous variables with a normal distribution, the median (P_25_, P_75_) for continuous variables with non-normal distributions, or as proportions for categorical variables. The chi-square test or Fisher exact test was used for 2-group comparisons of categorical variables. The Independent-Samples *t-*test was used for two-group comparisons of continuous variables with normal distributions. All tests were two sided with a significance level of at least 0.05.

## Results

### Clinical features and electrophysiological classification

A total of 48 patients (25 male, 23 female) were recruited, including 17 AIDP, 23 AMAN, and 8 equivocal. The mean age was 47.1 ± 14.7 years [range = 11–77 years, median = 47.5, interquartile range (IQR) = 35–59 years]. Probable or definite CBs were detected in 14 AMAN patients and 10 AIDP patients.

The summarization of existence of CBs in nerve segments of AIDP and AMAN patients was shown in Supplemental Table 1.

Logistic regression analysis showed no statistical significant correlation between reduction of Hughes grade at 1 month and age at onset, gender, or duration from onset to IVIG treatment.

### Comparison of clinical recovery in AMAN with and without CB

Table 1 showed clinical nadir and recovery in AMAN with and without CB, measured by Hughes grade. There was no difference in Hughes grade at nadir between the two groups. AMAN with CBs had higher reduction of Hughes grade at 1 month (1.71 ± 0.83 vs. 0.56 ± 0.73, *p* = 0.002), and higher percentage of patients with rapid recovery (64 vs. 11%, *p* = 0.029) than AMAN without CBs.

**Table 1:** Clinical nadir and recovery measured by Hughes grade in different types of GBS.

**Variables**	**AMAN with CB (*n* = 14)**	**AMAN without CB (*n* = 9)**	**AIDP with CB (*n* = 10)**	**AIDP without CB (*n* = 7)**	***P*****-values, 2-tailed probabilities**		
					**AMAN with vs. without CB**	**AIDP with vs. without CB**	**AIDP with CB vs. AMAN with CB**
H grade at nadir	3.43 ± 1.09	3.78 ± 0.83	3.3 ± 0.95	3 ± 0.82	*t* = 0.818	*t* = 0.678	*t* = 0.300
					*P* = 0.423	*P* = 0.508	*P* = 0.77
Reduction of H grade at 1 month	1.71 ± 0.83	0.56 ± 0.73	1 ± 0.67	1 ± 0.58	*t* = 3.437	*t* = 0.000	*t* = 2.257
					*P* = 0.002	*P* = 1	*P* = 0.034
Rapid recovery^*^/*n*	9/14 (64%)	1/9 (11%)	2/10 (20%)	1/7 (14%)	χ^2^ = 6.303	χ^2^ = 0.093	χ^2^ = 4.608
					*P* = 0.029	*P* = 1	*P* = 0.047
Slow recovery^#^/*n*	1/14 (7%)	2/7 (28%)	0/10	0/7	χ^2^ = 1.750	NA	NA
					*P* = 0.247		

*AIDP, acute inflammatory demyelinating polyradiculoneuropathy; AMAN, acute motor axonal neuropathy; GBS, Guillian-Barre syndrome; H grade, Hughes grade; CB, partial conduction block; SD, standard deviation; NA, not applicable*.

* *Rapid recovery: Improvement by two or more H grades at 1 month after onset*.

# *Slow recovery: H grade was 3 or more 6 months after onset*.

### Comparison of clinical recovery in AIDP with and without CBs

For patients with AIDP, there was no significant difference between those with and without CBs, in Hughes grade at nadir (3.3 ± 0.95 vs. 3 ± 0.82, *p* = 0.508), or reduction of Hughes grade at 1 month after onset (1.00 ± 0.67 vs. 1.00 ± 0.58, *p* = 1).

### Comparison of clinical recovery in AIDP with CBs and AMAN with CBs

As shown in Table 1, there was no difference in Hughes grade at nadir between AIDP with CBs and AMAN with CBs. AMAN with CBs had higher reduction of Hughes grade at1 month (1.71 ± 0.83 vs. 1 ± 0.67, *p* = 0.034), and higher percentage of patients with rapid recovery (64 vs. 20%, *p* = 0.047) than AIDP with CBs.

### Comparison of clinical recovery among different types of AMAN

There was no difference in Hughes grades at nadir among different types of AMAN. Patients with reversible CBs had the highest reduction of Hughes grade reduction at 1 month (2.14 ± 0.69 vs. 0.25 ± 0.50 in typical AMAN, vs. 1.5 ± 0.71 in AMAN with length-dependent CBs). None of typical AMAN patients showed rapid recovery, while 86% of those with reversible CBs showed rapid recovery.

## Discussion

In previous studies, patients with AMAN usually had a poorer or similar prognosis as compared with AIDP (6, 12, 13), but some patients with AMAN showed rapid recovery (14). Our results showed that the clinical severity before treatment was not related to CBs in either AIDP or AMAN group. Since clinical severity is also affected by other factors, including CB located at the proximal part (nerve root or plexus, which are difficult to define) and other electrophysiological changes, it might be difficult to obtain a simple positive relationship between clinical severity and CBs. We found positive correlation between CBs and good prognosis in AMAN, but not in AIDP. By comparing clinical improvement at 1 month between AMAN with CBs and AIDP with CBs, we found that AMAN with CBs showed better clinical improvement than AIDP with CBs. These results might be due to different pathophysiological mechanisms of CB in AIDP and AMAN.

In AIDP, demyelination impairs the transmission of impulses along nerve fibers by changing the properties of paranodal and internodal membranes. Therefore, it takes more current and a longer time to depolarize the node to threshold, resulting in an increased internodal conduction time. As demyelinating changes are serious enough, the current becomes insufficient to depolarize the node to threshold, resulting in CB (15). The pathophysiological mechanisms of CB, conduction velocity reduction and temporal dispersion are all due to demyelination. This could explain our result that, in AIDP, there was no significant difference between those with and without CB, in clinical nadir or clinical recovery.

In AMAN, infection by *C jejuni* bearing GM1-like or GD1a-like lipo-oligosaccharides may induce the production of IgG anti-GM1 or GD1a antibodies in certain patients. The autoantibodies bind to the nodes of Ranvier, where GM1 and GD1a are strongly expressed, and activate complement. Membrane attack complex is formed at the nodal axolemma, resulting in the disappearance of voltage-gated sodium (Nav) channels and the detachment of paranodal myelin loops. This pathophysiological changes may cause motor nerve CB, which might be potentially reversible. If autoimmune attack progresses with macrophages scavenging the injured axons, axonal damage and Wallerian degeneration develop (16, 17), and then CB disappeared. Thus, CB could be reversible or subsequentially followed by axon degeneration in AMAN. Our results showed that the functional grades at clinical nadir were similar in AMAN with CBs and without CBs. However, AMAN with CBs showed more rapid recovery and better clinical prognosis, with more reduction of Hughes grade at 1 month, and more patients with rapid recovery. Among the AMAN patients who had electrophysiological follow-up, seven had reversible conduction failure, and two had CB and subsequential axon degeneration. Those with reversible CB caused by sodium channel inactivation which could be resolved quickly without or with minimal structural changes following IVIG treatment, might be responsible for the good recovery.

The repairing of demyelination requires a much longer time than the resolving of sodium channel inactivation, which explains why AMAN with CBs showed better clinical improvement than AIDP with CBs by 1 month. The work of Tarek Sharshar et al. showed that, a proximal/distal CMAP ratio of the common peroneal nerve of above 56% was a good dichotomizing parameter to identify a group of GBS patients at very low risk for developing respiratory failure (7). This group of patients might be AMAN with reversible conduction failure.

Wang et al. reported that the severity of the illness was related to the development of CB in AIDP group and unclear classification group, but not in AMAN group (6). Both Wang et al. and Verma et al. found no relationship between CB and poor prognosis in all types of GBS (5). Our results are different from those reported by Wang and Verma (5, 6). We suspected that this might be due to different statistical methods applied. It would be better to analyze the correlation between CB and prognosis in more detailed subtypes, i.e., AMAN and AIDP, other than all patients with GBS. The latter might blur the correlation between CB and prognosis.

In conclusion, motor nerve CB is useful for prediction of functional outcome in GBS. CB, especially reversible CB, is a good prognostic factor in AMAN patients, but not in AIDP patients. Since the number of patients in our study was relatively small, especially the number of different types of AMAN patients, statistic comparison was unattainable among different types of AMAN (typical AMAN, AMAN with reversible CBs, and AMAN with length-dependent CBs). We expect more prospective studies to enroll more patients in the future, so as to better compare among different types of AMAN patients.

## Author's note

Part of this article was based on a poster presented at the AANEM Annual Meeting, Phoenix, Arizona, September, 2017.

## Ethics statement

This study was carried out in accordance with the recommendations of Peking Union Medical College hospital institutional review board with written informed consent from all subjects. All subjects gave written informed consent in accordance with the Declaration of Helsinki. The protocol was approved by the Peking Union Medical College hospital institutional review board.

## Author contributions

JN electrophysiology, acquisition of data, completion of statistical analysis, and drafting of the initial manuscript. QS electrophysiology, acquisition of data. YG, YL, SW, and QD electrophysiology. LC study concept and design, and critical revision of the manuscript for important intellectual content. ML study concept and design, data review, interpretation of results, revision of the initial draft, and writing of the final manuscript.

### Conflict of interest statement

The authors declare that the research was conducted in the absence of any commercial or financial relationships that could be construed as a potential conflict of interest.

## Abbreviations

AIDP: acute inflammatory demyelinating polyradiculoneuropathy
AMAN: acute motor axonal neuropathy
CB: conduction block
CMAP: compound motor action potential
EMG: electromyography
GBS: Guillian-Barre syndrome
IVIG: intravenous immunoglobulin
NCSs: nerve conduction studies.

## References

[1] Yuki N Hartung HP. Guillain-Barre syndrome. New Eng J Med. (2012) 366:2294–304. 10.1056/NEJMra1114525 22694000

[2] Rajabally YA Durand MC Mitchell J Orlikowski D Nicolas G. Electrophysiological diagnosis of Guillain-Barre syndrome subtype: could a single study suffice? J Neurol Neurosurg Psychiatry (2015) 86:115–9. 10.1136/jnnp-2014-307815 24816419

[3] Rajabally YA Hiew FL. Optimizing electrodiagnosis for guillain-barre syndrome: clues from clinical practice. Muscle Nerve (2017) 55:748–51. 10.1002/mus.25433 27750406

[4] Kokubun N Nishibayashi M Uncini A Odaka M Hirata K Yuki N. Conduction block in acute motor axonal neuropathy. Brain (2010) 133:2897–908. 10.1093/brain/awq260 20855419

[5] Verma R Chaudhari TS Raut TP Garg RK. Clinico-electrophysiological profile and predictors of functional outcome in Guillain-Barre syndrome (GBS). J Neurol Sci. (2013) 335:105–11. 10.1016/j.jns.2013.09.002 24064258

[6] Wang Y-C Feng G-D Wang J Liu X-D Zhao G Effect of conduction block in classification and prognosis of Guillain-Barre syndrome. Neuroimmunol Neuroinflamm. (2014) 1:77–81. 10.4103/2347-8659.139718

[7] Durand MC Porcher R Orlikowski D Aboab J Devaux C Clair B. Clinical and electrophysiological predictors of respiratory failure in Guillain-Barre syndrome: a prospective study. Lancet Neurol. (2006) 5:1021–8. 10.1016/S1474-4422(06)70603-2 17110282

[8] Asbury AK Cornblath DR. Assessment of current diagnostic criteria for Guillain-Barre syndrome. Ann Neurol. (1990) 27 (Suppl.):S21–4. 10.1002/ana.410270707 2194422

[9] Liu M Zou Z Guan Y Li J Zhou D Cui L. Motor nerve conduction study and muscle strength in newly diagnosed POEMS syndrome. Muscle Nerve (2015) 51:19–23. 10.1002/mus.24267 24752623

[10] American Association of Electrodiagnostic M Olney RK Guidelines in electrodiagnostic medicine. Consensus criteria for the diagnosis of partial conduction block. Muscle Nerve Suppl. (1999) 8:S225–9. 16921636

[11] Chan YC Punzalan-Sotelo AM Kannan TA Shahrizaila N Umapathi T Goh EJH. Electrodiagnosis of reversible conduction failure in Guillain-Barre syndrome. Muscle Nerve (2017) 56:919–24. 10.1002/mus.25577 28093784

[12] Ye Y Zhu D Wang K Wu J Feng J Ma D. Clinical and electrophysiological features of the 2007 Guillain-Barre syndrome epidemic in northeast China. Muscle Nerve (2010) 42:311–4. 10.1002/mus.21701 20589890

[13] Ye Y Wang K Deng F Xing Y. Electrophysiological subtypes and prognosis of Guillain-Barre syndrome in northeastern China. Muscle Nerve (2013) 47:68–71. 10.1002/mus.23477 23042578

[14] Ho TW Hsieh ST Nachamkin I Willison HJ Sheikh K Kiehlbauch J. Motor nerve terminal degeneration provides a potential mechanism for rapid recovery in acute motor axonal neuropathy after Campylobacter infection. Neurology (1997) 48:717–24. 10.1212/WNL.48.3.717 9065554

[15] Uncini A Yuki N. Electrophysiologic and immunopathologic correlates in Guillain-Barre syndrome subtypes. Expert Rev Neurotherapeut. (2009) 9:869–84. 10.1586/ern.09.43 19496690

[16] Bae JS Yuki N Kuwabara S Kim JK Vucic S Lin CS. Guillain-Barre syndrome in Asia. J Neurol Neurosurg Psychiatry (2014) 85:907–13. 10.1136/jnnp-2013-306212 24357682

[17] Willison HJ Jacobs BC van Doorn PA. Guillain-Barre syndrome. Lancet (2016) 388:717–27. 10.1016/S0140-6736(16)00339-1 26948435

