# Supplementary material for: Motor Nerve Conduction Block Predicting Outcome of Guillain-Barre Syndrome
Source: Front Neurol. 2018 Jun 1;9:399. doi: 10.3389/fneur.2018.00399 (PMC5992574; doi:10.3389/fneur.2018.00399)
Supplement: Supplementary file 1 [file Table_1.DOCX]

Supplement table 1. Distribution of CB in AIDP and AMAN patients

|  | CB | CB in AIDP | CB in AMAN |
| --- | --- | --- | --- |
| Median elbow-wrist | 14/80 | 3/80 | 11/80 |
| Median axilla-elbow | 1/80 | 1/80 | 0/80 |
| Ulnar below elbow-wrist | 6/80 | 2/80 | 4/80 |
| Ulnar above elbow-below elbow | 16/80 | 5/80 | 11/80 |
| Ulnar axilla-above elbow | 0/80 | 0/80 | 0/80 |
| Tibial popliteal-ankle | 6/80 | 1/80 | 5/80 |
| Peroneal below head-ankle | 24/80 | 10/80 | 14/80 |
| Peroneal above-below head | 4/80 | 0/80 | 4/80 |

*AIDP=acute inflammatory demyelinating polyradiculoneuropathy, AMAN=acute motor axonal neuropathy, CB=partial conduction block.*
